# What Is the Possible Minimum Death Rate?

**Published:** 1896-06

**Authors:** 


### What is the Possible Minimum Death Rate?

The late Dr. Parkes fixed 17 per 1,000 as the "mortality incident to human nature," and in his time—the infancy of hygiene and sanitation—even that figure seemed Utopian. But what shall it be now fixed at in view of the reduced rate in Greater London? In 1894 the death rate had fallen from 20.5 for the decennium, 1881-90, to 17.7 per 1,000; and last year, 1895, when the mean was 19.7, there were sanitary areas in the great metropolis with the following figures: Wandsworth, 14.8; Lee, 14.5; Lewisham, 14.4; Stoke Newington, 13.4, and Hampstead, 12.

